# Human type 2 17beta-hydroxysteroid dehydrogenase mRNA and protein distribution in placental villi at mid and term pregnancy

**DOI:** 10.1186/1477-7827-5-30

**Published:** 2007-07-10

**Authors:** Renée Drolet, Marc Simard, Julie Plante, Philippe Laberge, Yves Tremblay

**Affiliations:** 1 Ontogeny and Reproduction Unit, Centre Hospitalier Universitaire de Québec, Centre de recherche du CHUL, Canada; 2 Department of Obstetrics and Gynecology, Faculty of Medicine, Université Laval, Canada; 3 Centre de Recherche en Biologie de la Reproduction, Université Laval, Québec, Canada

## Abstract

**Background:**

During human pregnancy, the placental villi produces high amounts of estradiol. This steroid is secreted by the syncytium, which is directly in contact with maternal blood. Estradiol has to cross placental foetal vessels to reach foetal circulation. The enzyme 17beta-hydroxysteroid dehydrogenase type 2 (17beta-HSD2) was detected in placental endothelial cells of foetal vessels inside the villi. This enzyme catalyzes the conversion of estradiol to estrone, and of testosterone to androstenedione. It was proposed that estradiol level into foetal circulation could be regulated by 17beta-HSD2.

**Methods:**

We obtained placentas from 10 to 26 6/7 weeks of pregnancy from women undergoing voluntary termination of pregnancy, term placentas were collected after normal spontaneous vaginal deliveries. We quantified 17beta-HSD2 mRNA levels in mid-gestation and term human placenta by RT-QPCR. We produced a new anti-17beta-HSD2 antibody to study its spatio-temporal expression by immunohistochemistry. We also compared steroid levels (testosterone, estrone and estradiol) and 17beta-HSD2 mRNA and protein levels between term placenta and endometrium.

**Results:**

High 17beta-HSD2 mRNA and protein levels were found in both mid-gestation and term placentas. However, we showed that 17beta-HSD2 mRNA levels increase by 2.27 fold between mid-gestation and term. This period coincides with a transitional phase in the development of the villous vasculature. In mid-gestation placenta, high levels of 17beta-HSD2 were found in mesenchymal villi and immature intermediate villi, more precisely in endothelial cells of the stromal channel. At term, high levels of 17beta-HSD2 were found in the numerous sinusoidal capillaries of terminal villi. 17beta-HSD2 mRNA and protein levels in term placentas were respectively 25.4 fold and 30 to 60 fold higher than in the endometrium. Steroid levels were also significantly higher in term placenta than in the endometrium.

**Conclusion:**

The spatial and temporal expression of 17beta-HSD2 in the placenta during pregnancy and the comparison of 17beta-HSD2 expression and steroid levels between placental villi and endometrium are compatible with a role in the modulation of active and inactive forms of estrogens. Our observations strongly support the hypothesis that 17beta-HSD2 acts as a barrier decreasing estradiol secretion rates in the foetal circulation.

## Background

During human pregnancy, the placenta produces and secretes high amounts of estradiol (E2) and estrone (E1) [1,2]. E2, the most potent form, participates in the development of some foetal organ systems. Indeed, late in gestation, E2 cooperates in foetal lung maturation by stimulating Type II pneumocytes differentiation and surfactant phospholipid synthesis [3,4]. E2 also participates in activation of the hypothalamo-hypophyso-adrenal (HPA) axis [5,6] as well as in maturation of cutaneous barrier [7,8]. It is also well documented that maternal blood levels of estrogens rise continuously during all pregnancy [9-11]. However, the level of E2 in the umbilical vein supplying oxygenated blood to the foetus does not increase in parallel with that in the maternal vein, whereas the level of E1 in the umbilical vein continues to increase during pregnancy [12,13]. These asymmetric releases of E2 and E1 between foetal and maternal circulation strongly suggest the presence of a mechanism that controls the amounts of E2 entering into foetal circulation.

17β-hydroxysteroid dehydrogenases (17βHSDs) catalyze oxidoreduction at position C17 of C18 steroids such as E1 and E2, and C19 steroids (testosterone/androstenedione; DHEA/androstenediol) and at position C20 of progestins. The human placenta expresses mainly two 17βHSDs, types 1 and 2. Type 1 almost exclusively reduces E1 in E2 [14,15], whereas type 2 is reactive with E2 and testosterone with nearly comparable activities [16,17]. E2 synthesis in placenta depends on C19 steroid precursors dehydroepiandrosterone (DHEA) and its sulphate (DHEAS) from both maternal and foetal adrenal origin [1,2]. DHEAS is converted to DHEA by the sulfatase, then into androstenedione by the 3β-HSD type 1 (3β HSD1), then aromatized in E1 by the cytochrome P450 aromatase and finally reduced by the 17βHSD type 1 to generate E2.

In human placenta, 17βHSDs type 1 and type 2 are abundantly expressed [18,19]. All the enzymes required in E2 synthesis are exclusively expressed by the syncytial layer (SL) delineating the floating villi [14,18,20]. E2 is secreted in both maternal and foetal circulations. However, to reach the foetus, E2 must inevitably cross the endothelium that forms the wall of the blood vessels. We and others [18,21-23] have previously shown that in term placenta 17βHSD2 mRNA is exclusively expressed in the endothelial cells (EC) of foetal vessels [18]. In two separate ontogeny studies, 17βHSD2 protein was first detected around weeks 7 [21] or 12 of pregnancy [20]. Then, the number of 17βHSD2-positive cells increases, reaching a plateau around week 19 of gestation and staying at that level until term [14,18,20]. Another study has shown that 17βHSD2 is also detected in the chorionic vein at 19 weeks of pregnancy [22].

During normal placental development, villous trees expand extensively by sprouting [24]. Between weeks 15 and 21 of pregnancy, three different subtypes of villosities can be distinguished: the mesenchymal villi (MV), the immature intermediate villi (IIV) and the stem villi (SV). The MV is the forerunner of all other types of villi [24] and is in continuous formation during pregnancy [24,25]. During the first and the second trimesters the MV differentiates into IIV, and then IIV is transformed in SV. The MV tissue is rich in mesenchymal cells and poor in collagen. It is composed of loose connective tissues containing only few capillaries. The IIV also contains loose connective tissues, but is characterized by the presence of many arterioles and venules with only few undilated capillaries and an abundant stromal channel network [26]. By contrast, the SV contains a central vessel and is characterized by the presence of a stroma exhibiting huge and condensed bundles of collagen and by a well-developed paravascular capillary (PC) network beneath the SL [27]. During the last trimester of pregnancy, the MV differentiates into mature intermediate villi (MIV), which is then transformed into terminal villi (TV) [24], an highly efficient structure specialized in foeto-maternal exchanges. Indeed, TV contains highly dilated sinusoidal capillaries (Si) constituting more than 50% of the stromal volume. This the most abundant type of villi observed at term [25].

In the present study, we have further evaluated the mRNA and protein expression of the key steroidogenic enzyme 17βHSD2 in various structures of human placenta obtained at mid and term pregnancy. The levels of 17βHSD2 mRNA in several human placentas collected during this pregnancy interval have been precisely determined by real-time quantitative PCR using several housekeeping genes for data normalization. The protein has been detected by immunohistochemistry using a novel and very sensitive anti-17βHSD2 antibody, while the mRNA was localized by in situ hybridization. 17βHSD2 is recognized to play a role in the control of E2 levels in the endometrium [28,29]. To further evaluate the capacity of 17βHSD2 to regulate the amount of E2 entering into foetal circulation, we also have compared steroid levels (testosterone, estrone and estradiol) and 17βHSD2 mRNA and protein levels between the endometrium and the placenta. Taken together, our results support an important role for 17βHSD2 in the mechanisms that control estradiol levels in foetal circulation.

## Methods

### Human tissues

Human placentas from weeks 10 to 24 6/7 of pregnancy (n = 25) were obtained from women undergoing voluntary pregnancy termination. Term placentas (38–41 weeks of pregnancy, n = 10) were collected after normal spontaneous vaginal deliveries. No placenta had histopathological abnormality. Endometrial tissues in mid-late secretory phase were obtained by biopsy. For all patients, informed consent was obtained according to the policies for the Human Studies and the Institutional Review Board of the *Centre Hospitalier Universitaire de Québec *(protocols no 75-05-04, 70-05-05, 62-05-13, and 62-05-12). Gestational age was determined based on bi-parietal and abdominal diameters, and cephalo-caudal and femur lengths. Tissues were collected on ice. To avoid contamination of the villi by decidua or foetal membranes, a 0.5 cm slice of tissue was removed on each side of the placentas. Then, small pieces of villi (0.5 cm^3^) were prepared and extensively rinsed in PBS. Several pieces were kept frozen until RNA or protein extraction, while others were fixed in 4% paraformaldehyde for 24 hours prior processing for routine paraffin wax embedding. Five μm tissue sections were used.

### Northern blot analysis

Northern blot analysis were performed as described [18]. Briefly, 20 μg of total RNAs were glyoxalized and electrophoresed. After Northern blot, membranes were hybridized at 42°C and washed under high stringency conditions [18]. The 17βHSD2 full-length cDNA fragment [14], and the gamma-actin cDNA 2 kb fragment were used to prepare the probes [30].

### Real-time quantitative PCR

RNA extraction, cDNA synthesis, and real-time quantitative PCR were performed as described previously [31]. Briefly, total RNA extracts were prepared from 0.5 cm^3 ^placental samples and then purified on CsCl gradients. A 4 μg aliquot of total RNA was treated with DNase (DNase I, 0.25 unit/μg of total RNA), and reversed transcribed (Superscript II, Invitrogen) according to the protocol of the manufacturer, using hexameric random primer (pd(N)_6_, Invitrogen) in a 20 μl final volume. LightCycler-FastStart DNA Master SYBR Green I kits (Roche) were used for real-time PCR. Reactions were performed according to the protocol of the manufacturer with 0.5 μM of each primer (final concentration), 3 mM MgCl_2_, and an amount of cDNA samples corresponding to 100 ng of total RNA input in a 20 μl final volume. After enzyme activation (10 min, 95°C), 40 PCR cycles were performed: 0 sec, 95°C; 5 sec annealing temperature (see below); 20 sec, 72°C; 5 sec, temperature of fluorescence intensity reading (see below). At the end of each run, samples were heated to 95°C with a temperature transition rate of 0.2°C/sec to construct dissociation curves. Several PCR reactions were tested on a 2% agarose gel, and amplicons were subjected to DNA sequencing to confirm the specificity of the PCR reactions. The primers selected for 17βHSD2 encompass more than one intron. Gene/GenBank accession number/5' oligonucleotide/3' oligonucleotide/length of amplicon/annealing temperature/temperature of fluorescence intensity reading:17βHSD2/NM_002153/TGTCAGCAGCATGGGAGGAG/CGCCAAGATAGCATGCTGGA/346 nt/70°C/84°C; GAPDH/NM_002046/GAAGACTGTGGATGGCCCCTC/GTTGAGGGCAATGCCAGCCCC/358 nt/58°C/88°C; YWHAZ/NM_003406/AGACGGAAGGTGCTGAGAAA/GAAGCATTGGGGATCAAGAA/127 nt/59°C/76°C; TBP/NM_003194/GAACCACGGCACTGATTTTC/CCCCACCATGTTCTGAATCT/157 nt/58°C/80°C; HPRT1/NM_000194/TGACACTGGCAAAACAATGCA/GGTCCTTTTCACCAGCAAGCT/94 nt/59°C/78°C. A standard curve for real-time PCR was prepared for each gene using specific amplicons previously obtained by PCR, sequenced, and calibrated by electrophoresis on an agarose gel. The program supplied by the manufacturer (LightCycler Software, Version 3.5) was used to import the standard curves and calculate the amount of PCR products.

### Selection of normalization genes

To ensure a more accurate evaluation of the relative expression of 17βHSD2 mRNA in mid-gestation and term placentas, we assessed the expression stability of four housekeeping genes (GAPDH, YWHAZ, TBP, and HPRT1), based on the approach proposed by Vandesompele [32]. Housekeeping gene expression levels measured in all samples were evaluated with the geNorm program [32], which is a Microsoft Excel applet that estimates gene stability through numerous pair-wise comparisons. Following gene stability analysis, normalization factors were calculated from the expression levels of the three most stable housekeeping genes. Those were then used to normalize 17βHSD2 expression levels in the different placental samples. The same approach was used to determine housekeeping gene stability between term placentas and mid-late secretory phase endometrial tissues.

### Preparation and characterization of our novel anti-17βHSD2 polyclonal antibody

#### Polyclonal antibody against human 17βHSD2

A synthetic peptide (LEKDILDHLPAEVQ) corresponding to amino acids 279 to 292 of 17βHSD2 was synthesized by the *Service de Protéomique de l'Est du Québec *(CHUL Research Center) using standardized protocol, purified by HPLC, and coupled to keyhole limpet hemocyanin. A polyclonal anti-17βHSD2 antiserum was developed by repeated immunization of rabbits. The antiserum was purified using a sulfoLinK coupling gel (Pierce Biotechnology Inc., Rockford, IL).

#### Tissue fractionation and Western blot analysis

Villous tissues from term pregnancy were first fractionated in cytosol and microsomes as described previously [14,18]. Briefly, small pieces of villi were homogenized in ice-cold buffer containing 40 mM potassium phosphate (pH 7.0), 1.0 mM EDTA, and 20% (vol/vol) glycerol. Samples were then centrifuged at 1,000 × *g *for 10 min to remove cell debris and again at 105,000 × *g *for 1 h. Supernatants were saved as cytosol, and pellets as microsomes. Ten micrograms of each protein sample (cytosol and microsomes) were separated by SDS-PAGE and transferred to a nitrocellulose membrane (Bio-RAD). Membranes were blocked overnight at 4°C with 5% (wt/vol) fat-free dry milk powder in PBS containing 0,05% (vol/vol) Tween 20. These membranes were then incubated with our anti-17βHSD2 antibody (1:1000) for 2 hours at room temperature. After washing, bound antibodies were visualized with horseradish peroxidase-conjugated goat anti-rabbit antibodies using a Western Lightning kit (Perkin Elmer). An aliquot of the 17βHSD2 antiserum was pre-incubated at 4°C for 24 h with an excess (10^-6^M) of 17βHSD2 synthetic peptide used previously to develop the antibody. This adsorbed antiserum was used as negative control. The absence of primary antibody was also tested as negative control.

#### Two-dimensional gel electrophoresis

Total protein extracts were prepared from small pieces of term villi. Tissues were homogenized in PBS and then centrifuged 15 min at 250 × *g*. The pellet was re-suspended in 0.5% Nonidet P-40 in PBS. After a 15 min centrifugation at 300 × *g*, 9 volumes of acetone were added to the supernatant. After a 2 h incubation at -20°C, an aliquot was used for protein determination, and then 350 μg of proteins were centrifuged 15 min at 300 × *g*. The pellet was air dried and then dissolved in 7 M urea, 2 M thiourea (Sigma-Aldrich), 4% CHAPS (Sigma-Aldrich), 0.8% IPG buffer (Amersham Biosciences, Qc, Canada), bromophenol blue, and 10 mg/ml dithiothreitol (DTT). Denatured proteins were first separated according to their isoelectric point using 13 cm Immobiline DryStrip with a pH range of 3–10 with IPGphor isoelectric focusing system (Amersham Biosciences). Isoelectric focusing was done in four steps: re-hydration at 30 V for 10 h; 500 V for 1 h; 1,000 V for 1 h, and; 8,000 V to reach 17,500 Vh final. After these steps, strips were equilibrated 15 min in 50 mM Tris-HCl, 6 M urea, 30% (vol/vol) glycerol, 2% (wt/vol) SDS, and bromophenol blue. Then, each strip (total of two strips; samples were run in duplicate) was loaded on a 12% SDS-PAGE. After migration, proteins of one gel were silver stained while proteins of the second gel were transferred to a nitrocellulose membrane and 17βHSD2 protein was detected with our anti-17βHSD2 antibody as described above.

### Immunohistochemistry

Tissue sections were deparaffinized in xylene and rehydrated through graded ethanol. Tissue sections were incubated in 3% hydrogen peroxide in methanol for 30 min to block endogenous peroxidase activity and rinsed in distilled water. Antigen retrieval was carried out in sodium citrate buffer (0.1 M citric acid, 0.1 M sodium citrate) by boiling slides in a microwave oven for 2 min at maximum power and then cooling down at room temperature. Slides were rinsed once in distilled water for 5 min and once in PBS for 5 min before the blocking step. Unspecific binding was blocked by incubation of tissue sections in 10% goat serum in PBS containing 0,1% BSA and 0,4% Triton X-100 for 30 min. The tissue sections were then incubated with primary antibodies overnight at 4°C in a humidified chamber. The anti-17βHSD2 antibody was used at 1:200 dilution, the anti-KDR (Chemicon Internationnal, Temecula, CA) was used at 1:50 dilution. KDR/VEGF-R2 (Vascular endothelial growth factor – Receptor 2) was used as an endothelial cell marker. Expression of KDR decreases as pregnancy progress and becomes barely detectable near term. Following the incubation with the primary antibody, the slides were washed 3 times in PBS and incubated with biotinylated goat anti-rabbit (DakoCytomation Inc. Mississauga, Ontario, Canada) for 17βHSD2 and goat anti-mouse (Chemicon International Inc., Mississauga, Ontario, Canada) for KDR for 45 min at room temperature. The slides were washed 3 times in PBS and the antigen-antibody complex was revealed using avidin-biotin peroxidase reaction method using the ABC Vectastain kit (Vector Laboratories, Inc., Burlingame, CA) and AEC (3-Amino-9-ethylcarbazole) as chromagen. The immunoreactivity was visualized as red color. Control experiments for the 17βHSD2 were performed on adjacent sections by substitution of the antibody by the pre-immune rabbit serum or by the 17βHSD2 antibody adsorbed with an excess (10^-6 ^M) of the synthetic peptide instead of primary antibody. Anti-KDR control experiments were performed by replacing the primary antibody with 0.01 M PBS.

### Immunohistochemistry quantification

Quantification of 17βHSD2 signal in different parts of mid-gestation and term placentas were performed using a Zeiss Axioskop 2 Plus microscope linked to a digital camera (Spot Insight, Carsen Medical Scientific, Markham, ON, Canada) using Image Pro-Plus software (Carsen Medical Scientific). Red staining intensities in IIV and SV from mid-gestation placentas or SV and TV from term placentas were quantified as follows using Image Pro-Plus. Briefly, IIV, SV or TV were delimitated using the "New AOI" command. The created areas of interest were measured using measurement commands. The "Select Color" command was used to specify the range of intensities/colors that defined the section analysed. The selected range color was applied for each measurement. The ratio of the mean intensity/density values was calculated for each type of villi from the same slide. Twenty-three IIV and 17 SV structures were analysed from 13 mid-gestation placentas. For the analyses on term placenta, 10 TV and 9 SV structures were analysed from 5 placentas.

### In situ hybridization

Synthesis of RNA probes and in situ hybridization were performed as described previously [18,33] with minor modifications. Briefly, RNA probes were synthesized from the 303 bp *Eco *RV fragment of 17βHSD2 [34] inserted into pSV-SPORT-1. After linearization of the plasmid DNAs by *Bam *HI and *Xho *I, antisense and sense 17βHSD2 RNA probes were synthesized using (^35^S)-UTP (NEN Life Science Products, Boston, MA). Probes were synthesized using the Riboprobe Combination System kit (Promega Corp., Madison, WI), and riboprobes with less than 1.4 × 10^9 ^dpm/μg DNA matrix were discarded. After deparaffinization and re-hydration, tissue sections were fixed in 4% paraformaldehyde (Sigma, St-Louis, Missouri USA) for 20 min, treated with proteinase K (10 μg/ml; Sigma) for 25 min at 37°C, acetylated in 37.5 mM triethanolamine solution (Sigma) containing 0.25% (vol/vol) anhydric acid (Sigma) for 10 min, dehydrated in graded alcohol solutions, and air-dried. Hybridization (2 × 10^6 ^dpm/100 μl/slide) was performed overnight at 58°C in 50% formamide, 0.3 M NaCl, 10 mM Tris-HCl (pH 8.0), 1 mM EDTA, 1× Denhardt's (100× = 2% BSA, 2% polyvinylpyrrolidone, and 2% Ficoll), 1% dextran sulphate, 10 mM DTT, and 500 μg/ml transfer RNA. After hybridization, slides were treated with ribonuclease A for 30 min and washed under high stringency conditions in 0.1× SSC (Standard Saline Citrate) and 1 mM DTT at 58°C for 30 min. After defatting, tissues were coated with NTB-2 emulsion (Eastman Kodak Co., Rochester, NY) and kept at 4°C for 5 to 10 days. Slides were developed with D-19 solution (Eastman Kodak Co.), counterstained with 0.25% thionine (Sigma), dehydrated, and mounted with Permount (Fisher Scientific).

### Western blot analysis of 17βHSD2 protein in human term placenta and in mid-late secretory phase endometrial tissue

Placental and endometrial tissues were homogenized in ice-cold buffer containing 10% SDS, 10 mM Tris-HCL pH 7.5, 1 mM DTT and 1 mM PMSF. Proteins were precipitated with methanol-chloroform, solubilized in SDS-PAGE sample buffer containing 1% SDS, 62.5 mM Tris-HCL pH 6.8, 1% 2-mercaptoethanol, 10% glycerol and 0.025% bromophenol blue, heated at 95°C for 3 min then stored at -20°C until use. Protein concentration of samples was determined by BCA protein assay after precipitation with trichloro acetic acid. 50 μg of endometrial proteins and 50, 2.5, 1.0, 0.5, 0.25, and 0.1 μg of placental proteins were separated by SDS-PAGE and transferred to a nitrocellulose membrane (Bio-RAD) for Western blot analysis. Membranes were treated as described above. After washing, bound antibodies were revealed by chemiluminescence with horseradish peroxidase-conjugated goat anti-rabbit IgG antibodies using a SuperSignal WestDura kit (incubation 5 min) and Kodak XAR films (exposition 30 sec).

### Measurement of testosterone, estrone and estradiol in human term placenta and in mid-late secretory phase endometrial tissue

Steroid hormones were extracted as follows. Frozen tissues (200 mg) were pulverized, weighed, and transferred into glass tubes. After suspension in 0.5 ml PBS pH 7.0, 1 ml of ethanol:acetone (1:1, v:v) was added. Samples were homogenized and centrifuged to pellet tissue debris. Supernatants were exposed to nitrogen gas flow to remove organic solvents and the volumes were measured by a gas chromatographic mass spectrometric method developed to measure steroid hormone levels in rat and monkey sera [35]. Briefly, steroids were extracted from PBS by liquiq-liquid and solid-phase extraction. Derivatization reactions were performed to improve chromatographic and detection responses. Testosterone, estrone and estradiol were quantified by gas chromatography and negative chemical ionization mass spectrometry (GC/MS) as reported [35].

### Statistical analysis

Statistical analyses were performed usingt-test function of GraphPad Prism 3.0 (GraphPad Software, San Diego, CA) and SPSS 10.0.5 softwares (SPSS, Chicago, IL). A two-tailed *p *value of less than 0.05 was considered a significant difference.

## Results

### Levels of 17βHSD2 mRNA in different sections of the human term placenta

**Figure 1:**
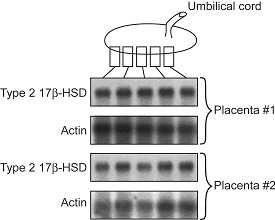
Northern blot analysis of five samples of villous tissue collected at different positions relative to the insertion point of the umbilical cord using 20 μg of total RNA loaded in each lane. Blots were probed with 17βHSD2 and actin cDNAs. Two placentas obtained at term were studied. Autoradiograms for Northern blots were densitometrically scanned and no significant variation between samples was observed.

17βHSD2 mRNA levels were determined by Northern blot analysis in 5 samples of villi collected at different positions relative to the insertion point of the umbilical cord. Two placentas obtained at term pregnancy were studied. 17βHSD2 mRNA levels in placental samples are quite similar, and independent from the sampling site (Figure 1).

### Levels of 17βHSD2 mRNA in human placenta during pregnancy

**Figure 2:**
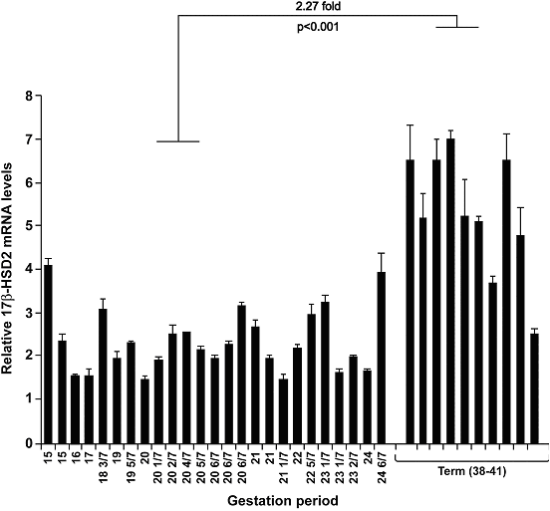
Expression of 17βHSD2 mRNA in human mid-pregnancy and term placentas. Levels of 17βHSD2 mRNA were determined by real-time quantitative PCR in 35 placental tissues collected at the indicated gestation time. For each sample, 17βHSD2 expression level was normalized by a factor generated from the expression levels, in the same sample, of the three most stable housekeeping genes studied (YWHAZ-HPRT1, and GAPDH), using the geNorm software (for details, see Methods). Means ± SD are presented.

17βHSD2 mRNA levels were determined by real-time PCR in 25 samples obtained between gestation weeks 15 and 24 6/7 and in 10 samples obtained at term (38–41 weeks). No significant variation was observed in the expression of the 17βHSD2 among samples collected around mid-gestation (Figure 2). However, a 2.27 fold increase was observed in the group of samples collected at term when compared to the group of samples collected at mid-pregnancy. No difference in 17βHSD2 mRNA expression according to foetal sex was observed (data not shown).

### Characterization of the anti-17β-HSD2 polyclonal antibody

**Figure 3:**
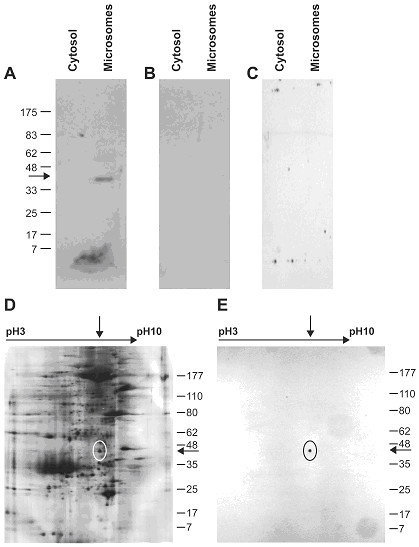
Characterization of the anti-17βHSD2 antibody. Western blot analysis: Cytosol and microsomes extracted from human placental villi were resolved on a 12% SDS-PAGE and transferred on nitrocellulose membrane. Immunodetection was performed using purified rabbit anti-17βHSD2 antibody (*Panel A*), preimmune rabbit serum (*Panel B*), or antibody pre-adsorbed with an excess (10^-6^M) of 17βHSD2 synthetic polypeptide (*Panel C*). Specificity of the anti-17βHSD2 antibody was also studied by two-dimensional gel electrophoresis (*Panels D and E*). Microsomal fraction of placental villi was separated by 2-dimensional isoelectric focusing-SDS-PAGE. Panel D, silver staining. Panel E, immunodetection with the purified anti-17βHSD2 antibody. Position of the circle on panel D corresponds to the location of the circle on panel E. The upper arrow corresponds to a pI value of approximately 8 to 9 and arrow on the right side corresponds to the expected molecular weight.

The specificity of our novel anti-17βHSD2 polyclonal antibody was evaluated by Western blot, two-dimensional gel electrophoresis, and immunohistochemistry. In Western blot, a single positive signal of Mr 42000 was detected in placental microsome cell fraction but not in cytosol, as expected for 17βHSD2 [14] (Figure 3A). There was no signal when pre-immune serum was used instead of the antibody (Figure 3B) or when the antibody was first adsorbed with an excess of the 17βHSD2 synthetic polypeptide (Figure 3C). Finally, only one positive signal was detected with this antibody from a microsomal fraction of placenta resolved by two-dimensional gel electrophoresis (Figure 3D and 3E). Immunohistochemistry was also performed on endometrial tissue and the staining was specific to glandular epithelial cells of the secretory endometrium, as reported previously [29] (data not shown). We conclude that our antibody specifically detects the 17βHSD2 protein.

### Immunohistochemistry of 17βHSD2 and KDR in human placenta at mid-gestation

**Figure 4:**
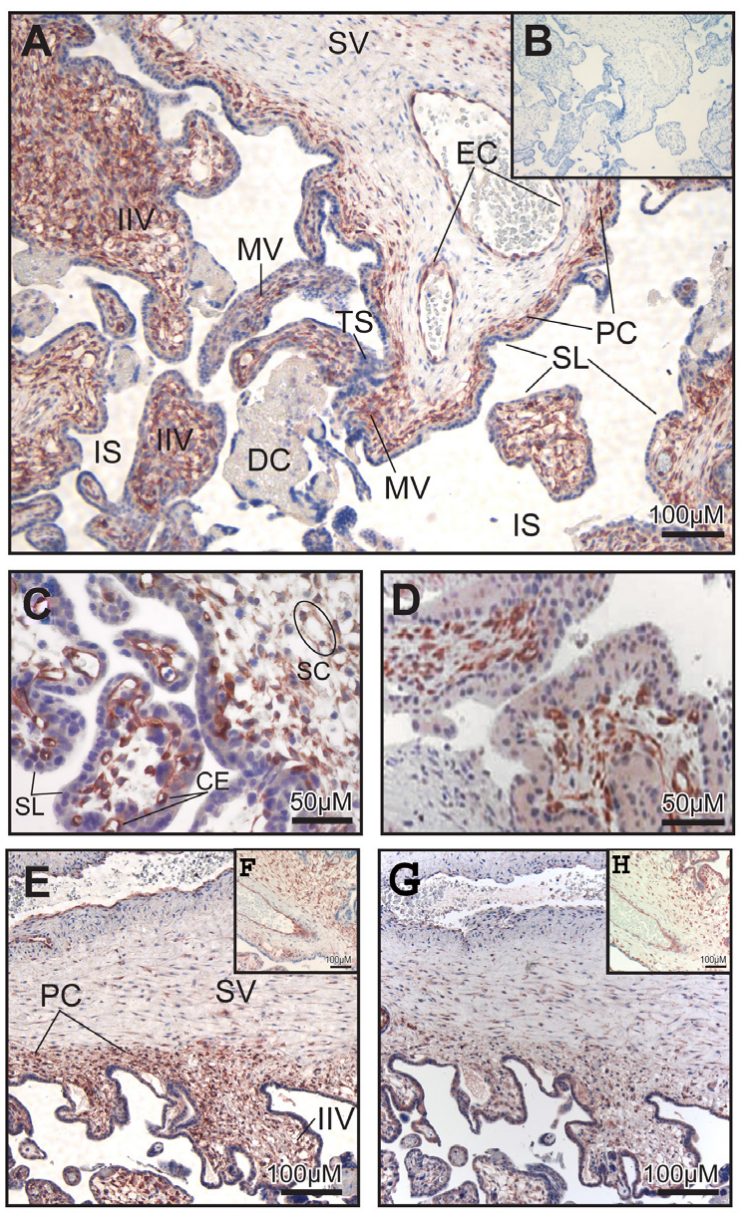
Localization of 17βHSD2 and KDR proteins in mid-gestation placentas. Tissue sections of human placentas obtained at 19 weeks (*Panels A and B)*, 18 weeks (*Panel C*), 10 weeks *(Panel D*) or 20 weeks (*Panels E-H*) of pregnancy were immunostained with anti-17βHSD2 (*Panels A, C-F*), with pre-adsorbed antibody, as negative control (*Panel B*), or with anti-KDR antibody used as endothelial cell marker (*Panels G and H*). 17βHSD2 protein expression is observed in immature intermediate villi (IIV) (*Panels A, C, E, F*), in capillary endothelial (CE) cells and in cells forming the stromal channel (SC) (*Panel C*). Positive cells (most probably endothelial cells (EC)) were found in mesenchymal villi (MV). No signal was observed in trophoblastic sprout (TS), in syncytial layer (SL), and in decidua (DC) (*Panel A*). In stem villi (SV) (*Panel A*), EC of large blood vessels and those of paravascular capillary (PC) network are positive. Endothelial cells forming PC network in SV (*Panel G*) and those composing the wall of the stromal channel were immunostained by the anti-KDR (*Panel H*). Intervillous space (IS), where maternal blood circulates, is indicated.

At mid-gestation, the placenta is mainly composed of mesenchymal villi (MV), immature intermediate villi (IIV) and stem villi (SV). These three types of villi are shown in Figure 4A. Early in pregnancy (10 weeks), 17βHSD2 was easily detected in mesenchymal cells of the MV (Figure 4D). At mid-gestation, a majority of cells composing the stroma in the MV were 17βHSD2-positive (Figure 4A). In the IIV, staining for 17βHSD2 was very strong and uniform (Figure 4A). The stroma of the IIV is composed of longitudinally oriented stromal channels (SC) (Figure 4C), and immunoreactivity was observed in the majority of the cells composing the channel's periphery. In the IIV, several capillaries are present, particularly beneath the syncytial layer. The endothelial cells of the foetal capillaries in the IIV strongly expressed the 17βHSD2. When the IIV is transformed into SV, the transition begins with a stromal fibrosis starting around a centrally positioned foetal vessel [24]. The SV at mid-gestation is mainly composed of 1 or 2 central foetal vessels and a paravascular capillary network (PC) beneath the syncytial layer (Figure 4A). The staining for 17βHSD2 was very strong in endothelial cells of the capillaries forming the PC. Endothelial cells of the foetal vessels (EC) also expressed the 17βHSD2. Cells forming the PC network in SV showed a positive signal for KDR (Figure 4G). In IIV, both endothelial cells of the capillary network and cells composing the wall of stromal channel were immunostained by the anti-KDR (Figure 4H).

### Immunohistochemistry of 17βHSD2 in human term placenta

**Figure 5:**
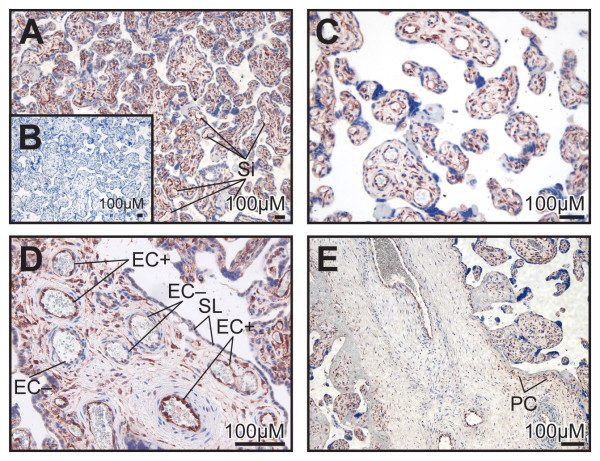
Localization of 17βHSD2 protein in human term placentas. Tissue sections of term placentas were immunostained with anti-17βHSD2 (*Panels A, C-E*) or with pre-adsorbed antibody, as negative control (*Panel B*). Strong positive signals were observed in terminal villi (TV) (*Panels A and C*), more precisely in sinusoidal capillaries (Si). 17βHSD2 expression is observed in the paravascular capillary net (PC) as well as in endothelial cells (EC) forming the blood vessel walls of stem villi (SV) (*Panels D and E*). Some blood vessels are EC positive (+) and others EC negative (-).

At term, the placental villi is mainly composed of terminal villi (TV) and stem villi (SV). TV is the most abundant type of villi at term, with more than 50% of its stromal volume occupied by the foetal capillaries. All these capillaries strongly expressed the 17βHSD2. Again, the syncytium was negative. In the SV (Figure 5D and 5E), 17βHSD2 was localized in endothelial cells of the foetal blood vessels and in capillaries composing the PC. However, some large blood vessels were clearly and strongly positive for 17βHSD2 (EC+), while others showed a signal close to the background (EC-) (Figure 5D).

### Localization of 17βHSD2 mRNA in mid-gestation and term placenta by in situ hybridization

**Figure 6:**
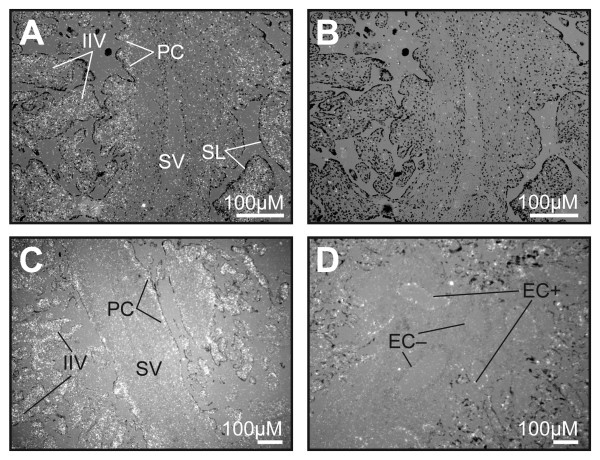
Localization of 17βHSD2 mRNA in human mid-pregnancy and term placentas. In situ hybridization was performed with antisense (*Panels A, C and D*) and sense (negative control, *Panel B*) RNA probes on tissue sections of placentas obtained at 18 weeks of gestation (*Panels A, B, and C*), or at term (*Panel D*). Emulsion granules (positive signals) appear as white dots on darkfields. SV, stem villi; IIV, immature intermediate villi: SL, syncytial layer; PC, paravascular capillary net; EC+ and EC-, endothelial cells with, respectively, positive and negative signal with the 17βHSD2 antisense probe.

In mid-gestation placentas, 17βHSD2 mRNA was abundant in the IIV and in the PC network of the SV (Figure 6A and 6C). The mRNA was also detected in endothelial cells of large blood vessels in SV (Figure 6D). At term, in situ hybridization revealed that 17βHSD2 mRNA was abundant in TV (data not shown). This result concurs with that obtained by immunohistochemistry (Figures 4 and 5). In the SV, expression of the 17βHSD2 gene was detected in endothelial cells of several large blood vessels (EC+). As observed in immunohistochemistry experiments, the staining in some vessels was close to the background (EC-).

### Quantitative evaluation of 17βHSD2 immunohistochemistry staining

**Figure 7:**
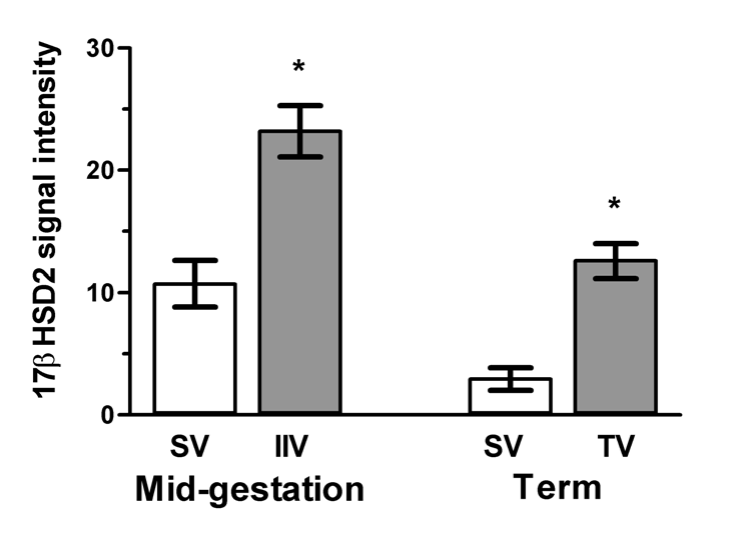
Quantitative evaluation of 17βHSD2 protein expression in immature intermediate villi (IIV) and stem villi (SV) of human mid-gestation placentas and from terminal villi (TV) and stem villi (SV) of human term placentas. Twenty-three IIV and 17 SV structures were analysed from 13 mid-gestation placentas, while 10 TV and 9 SV structures were analysed from 5 term placentas. Quantification of immunohistochemical stainings was performed using a Zeiss Axioskop 2 plus miscroscope linked to a digital camera (Spot Insight; Carsen Medical Scientific, Markham, ON, Canada) using Image Pro software analysis (Carsen Medical Scientific). *: *p *< 0.001.

Quantification of 17βHSD2 staining using the Image-Pro Plus software showed that in mid-gestation placenta, 17βHSD2 was more abundant in the IIV than in the SV and that in term placenta, 17βHSD2 was more abundant in the TV than in the SV (Figure 7).

### Estrone, estradiol and testosterone levels, and 17βHSD2 mRNA and protein levels in term placenta and mid-late secretory phase endometrial tissue

As shown in Table 1, estrogens levels measured in term placenta are much higher than in endometrial tissue. Also, in term placenta, level of testosterone is very low when compared to those of E1 and E2. However, in both tissues, E1 and E2 levels are quite similar. We also compared mRNA and protein levels of 17βHSD2 between term placenta and endometrium. 17βHSD2 mRNA level, as determined by QPCR, and 17βHSD2 protein level, as determined by Western blot, were respectively 25.4 fold and 30 to 60 fold higher in the placenta than in the endometrium (Table 1).

**Table 1:** 17βHSD2 expression ratios and steroid determinations in term placenta and in endometrium during mid-late secretory phase

17βHSD2 expression ratios (placenta/endometrium)		Steroid levels			
**mRNA**	**protein**		**T**	**E1**	**E2**

		Placenta (ng/g ± SD)	1.57 ± 1.51	52.64 ± 9.72	39.66 ± 12.76
25.4 ± 5.8	30–60^a^	Endometrium (ng/g ± SD)	n/a	0.064 ± 0.026	0.052 ± 0.021
		Ratio placenta/endometrium	n/a	827 ± 376	751 ± 388

^a^This range of variation was estimated from comparison of the densitometric values obtained among endometrial samples and a series of dilutions of placental samples in Western blot experiments.

## Discussion

During pregnancy, E2 plays an important role in the maturation of some foetal organs such as the lungs, skin and HPA axis [3-7,36]. However, excessive exposure to estrogenic compounds could have deleterious consequences on foetal development. Indeed, clinical reviews of women who were exposed to the synthetic estrogen diethylstilbestrol showed that foetal exposure to high estrogenic compound had adverse developmental effects and could result in anomalies in adulthood [37-44]. Exposure *in utero *to environmental components with estrogenic activity could also results in reproductive disorders [45-47]. Elevated levels of natural estrogens during gestation have been associated with an increase in children breast or testicular cancer [48-50]. A study conducted in rat has shown that minor elevation of E2 level in gestation can retard foetal development or be lethal [51]. It was hypothesized that entry of E2 in foetal circulation may be limited by presence of 17βHSD2 in foetal endothelial cells [18,20,22]. In support to this, E2 increase in maternal vein during pregnancy is not accompanied by a parallel increase in the umbilical vein, while E1 level increases during pregnancy in the umbilical vein [9,10,12,13]. This study of 17βHSD2 expression in placenta was designed to assess the potential of this enzyme to act as a barrier in the control of E2 transfer in foetal circulation during pregnancy.

17βHSD2 mRNA level was measured in different parts of term placenta to evaluate if there is a spatial variation in expression (Figure 1). Our results indicate that 17βHSD2 mRNA expression is independant of the sampling site. Also, this supports the concept that 17βHSD2 should be expressed at similar levels throughout placental villi to assume an efficient role in the control of the amount of E2 secreted into foetal circulation.

We have precisely determined the level of 17βHSD2 mRNA by real-time quantitative PCR in different samples of mid-gestation and term placentas (Figure 2). 17βHSD2 mRNA levels were quite stable between 15 and 24 weeks, whereas 17βHSD2 mRNA levels increased by 2.27 fold between mid-gestation (15–24 weeks) and term (38–40 weeks). The period between mid-gestation and the beginning of the third trimester is an important transitional phase in villous development [24,26], characterized by expansion of the foetal capillary bed and branching angiogenesis. Between 10–13 weeks and term, the proportion of villous volume occupied by the capillaries increases from 6% to 25%, the major change occurring at 23–24 weeks [52]. In contrast, the proportion of villous volume occupied by the trophoblasts remains relatively constant throughout pregnancy [52]. Since 17βHSD2 is expressed in endothelial cells, the observed increase in mRNA levels is in agreement with the developmental pattern of the placental vasculature.

We have characterized the distribution of 17βHSD2 in placental villi at different developmental stages using immunohistochemistry (Figures 4 and 5). Quantitative evaluation of 17βHSD2 protein-positive staining in placental structures shows that in mid-gestation placenta, 17βHSD2 is more abundant in immature intermediate villi (IIV) than in stem villi (SV). It also shows that at term, 17βHSD2 is more abundant in terminal villi (TV) than in stem villi (SV) (Figure 7). By in situ hybridization, strong signals were obtained in both mid-gestation IIV and term TV (Figure 6). In the mid-gestation mesenchymal villi (MV), the majority of cells composing the stroma were positive for 17βHSD2 (Figure 4). MV is the forerunner of all types of villi and is transformed into IIV at mid-pregnancy [24]. This transition is characterized by an expanded loose stroma and an increase in capillary density due to branching angiogenesis [26]. IIV is the prevailing villous type until the end of the second trimester [24]. In IIV, 17βHSD2 is very abundant in the majority of cells forming the edge of the stromal channel, and in cells forming the capillary network (Figure 4). The enzyme is also present in endothelial foetal capillary cells. At mid-gestation, cells forming the edge of the stromal channels express several angiogenic factors like vascular endothelial growth factor-A (VEGF-A), angiopoietin-1, and angiopoietin-2 [53] that are involved in the development and remodelling of placental vasculature [26,54]. Also, it was reported that KDR (VEGF-R2) is expressed by endothelial cells of foetal vessels and by endothelial cell precursors [55]. KDR was also localized in cytotrophoblasts [56]. We observed a correlation between 17βHSD2 and KDR stainings (Figures 4E and 4G, and 4F and 4H), thus suggesting that positive cells in the stroma of MV and IIV should be endothelial cells or endothelial cell precursors. It is interesting to note the presence of KDR in cytotrophoblasts, while no staining was detected for 17βHSD2 in those cells. In SV, staining for 17βHSD2 was very strong in endothelial cells of PC network underneath the syncytial layer, and in endothelial cells of mature venules and arterioles. However, the stroma was negative. In term placenta, 17βHSD2 is strongly expressed in the numerous foetal capillaries of TV (Figure 5), which is the most effective structure for foeto-maternal exchanges. Indeed, capillaries make up more than 50% of its stromal volume. Our results show that in both mid-gestation and term placenta, 17βHSD2 protein is concentrated in structures were exchanges between the mother and the foetus are the most effective.

With respect to E2 inactivation, it is interesting to compare 17βHSD2 mRNA and protein, and steroid levels between the placenta and the endometrium. In endometrium, 17βHSD2 is expressed in glandular epithelial cells during the mid to late secretory phase [17,29]. Several studies showed that 17βHSD2 is absent in secretory endometriotic tissues [17,28,29]. The absence of 17βHSD2 expression favors the increasing quantities of E2 observed in endometriosis [17,29]. We show that levels of 17βHSD2 mRNA and protein are 25.4 and 30 to 60 fold higher, respectively, in term villi than in mid-late secretory phase endometrium (Table 1), thus supporting an important capacity of E2 inactivation by the placenta. As also shown in Table 1, the placental tissue contains much higher levels of estrogens than endometrial tissue. However, the levels of E1 and E2 were similar in each tissue, suggesting that 17βHSD2 favors equilibrium between inactive and active forms of estrogens, and that elevated levels of E2 in placenta do not overwhelm the inactivating capacity of the enzyme. Also, the measured levels of testosterone (T) and E2 in term placenta are 1.57 ng/g and 39.66 ng/g, respectively, suggesting that E2 is the principal substrate of 17βHSD2, since both steroids have a similar affinity for the enzyme. Taken together, these observations support the concept that levels of 17βHSD2 in the human placenta are sufficient to yield significant rates of conversion of E2 into E1, despite the fact that placental E2 concentrations are elevated.

In conclusion, the spatial and temporal expression of 17βHSD2 mRNA and protein in the placenta during pregnancy and the comparison of 17βHSD2 expression and steroid levels between term placenta and mid-late secretory phase endometrium provide clear evidences that 17βHSD2 can act as a barrier to protect the foetus against excessive E2 levels.
